# Conservative Treatment of a Large Facial Midroot Perforation

**DOI:** 10.1155/2015/326302

**Published:** 2015-03-09

**Authors:** Stephane Kerner, François Bronnec

**Affiliations:** ^1^Department of Periodontology, Service of Odontology, Rothschild Hospital, AP-HP, UFR of Odontology, Paris 7-Denis Diderot University, 5 rue Santerre, 75012 Paris, France; ^2^Department of Conservative Dentistry and Endodontics, Pitie-Salpetriere Hospital, AP-HP, UFR of Odontology, Paris 7-Denis Diderot University, 47/83 boulevard de l'Hopital, 75013 Paris, France

## Abstract

*Aim*. To report on the endodontic and periodontal management of a root and alveolar process perforation in a maxillary front tooth. *Summary*. Perforation during access cavity preparation is an infrequent complication during endodontic therapy, leading to potential periodontal tissue breakdown. The case described the two-stage management of a massive facial root perforation requiring a connective tissue graft to correct a mucosal fenestration persisting after orthograde repair of the root defect with MTA.

## 1. Introduction

Root canal perforation is defined as a communication between the root canal system and the periodontal attachment apparatus through the root canal walls. Iatrogenic root perforation is a procedural mishap but quite an uncommon event [1], which may occur at any stage of root canal therapy, from access cavity to postspace preparation, on any tooth [2, 3]. Besides the direct damage to root structure and its consequences in terms of mechanical weakening of the tooth, one must consider this first as a traumatic injury to the periosteum and second as a potential pathway from microorganisms [4]. As opposed to pathological root resorption defect, accidental root perforation raises a significant therapeutic challenge, because if not properly treated without delay, the breakdown of periodontal tissue may lead to the formation of a periodontal pocket.

Furthermore, perforation has been shown to negatively impact the outcome of nonsurgical endodontic treatment [5].

Both management and prognosis are associated with the location of the tooth in the arch, the level of the perforation, the side of the root that is affected, the size of the defect, and the time. Several materials and technics have been used earlier to address this kind of problem either surgically [6, 7] or nonsurgically [8, 9].

Presented as a root end filling material in 1993 [10] and first available under the brand name ProRoot MTA, calcium silicate based cements have been used for orthograde and surgical repair of root perforations [11, 12]. Due to its unique properties of hard setting in the presence of moisture [13] and proven biocompatibility [14], it is now the biomaterial of choice to effectively seal any communication between the root canal system and the periosteum when traditional root filling methods are contraindicated. Successful treatment of root perforations has been reported with the use of MTA in both short- and long-term clinical studies [15, 16].

Depending upon the tooth being affected and the localization of the perforation defect along the root surface, an esthetic defect can arise, especially in maxillary front teeth.

This paper presents the multidisciplinary management of a buccal, midroot perforation on a maxillary central incisor complicated with a mucosal fenestration that needed a surgical correction after orthograde repair.

## 2. Report

**Figure 1:**
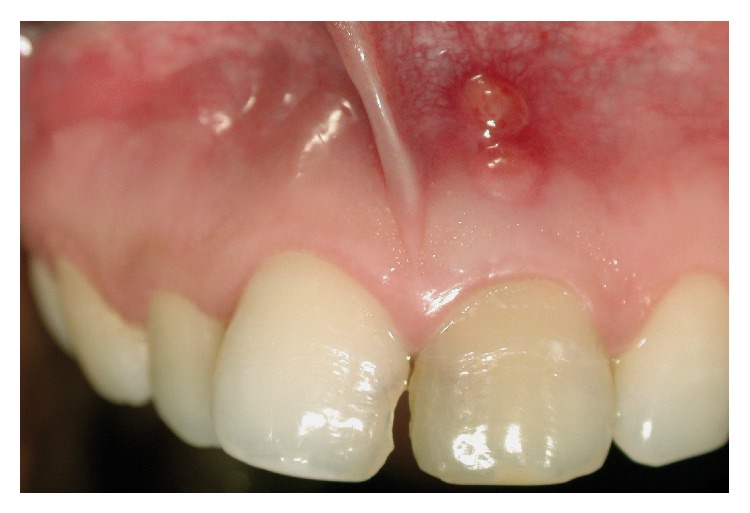
Clinical view of the two fistulas on tooth 21.

A 20-year-old Chinese female patient presented to the Department of Conservative Dentistry and Endodontics with a history of a nonsurgical root canal treatment on the left maxillary central incisor (tooth 21 using the FDI World Dental Federation Two-Digit Notation) the week before. She mainly complained about the recent development of two fistulous tracts in the upper arch (Figure 1).

The medical history was noncontributory; she had good oral hygiene and a normal healthy gingiva except at the buccal surface adjacent to the involved tooth 21.

Teeth 11 and 21 presented with deep cavities; all teeth in the area responded within normal limits to cold test except for tooth 21 which showed an access cavity filled with a temporary dressing on the palatal surface of the crown. The probing of the gingival sulcus was normal for each tooth without pocketing, whereas tooth 21 was slightly responsive to percussion.

**Figure 2:**
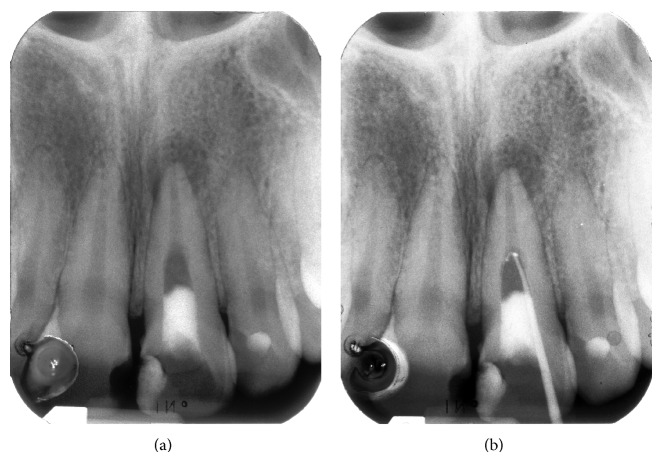
(a) Radiograph of tooth 21. (b) A gutta-percha point inserted in fistulous tract showing the midroot facial perforation.

A radiograph taken with a film holder (XCP film holder, Dentsply Rinn, Elgin, Illinois) showed extensive coronal decay on both teeth 11 and 21 and a radiolucency superimposed on the canal outline altering it (Figure 2). Another radiograph taken with a gutta-percha point (Autofit non-standardized MF gutta-percha cone, Sybron Endo, Orange, California) tracing the sinus tract demonstrated the origin of the coronal sinus being a large buccal midroot perforation on tooth 21 while the other fistulous tract originated from the periapical lesion of the same tooth.

Careful removal of carious tissues was performed under rubber dam isolation after disinfection of the operative field and the tooth (0.12% chlorhexidine digluconate solution, hospital facilities). The cavities on teeth 11 and 21 were then filled with a direct composite restoration (EsthetX, Dentsply Caulk, Milford, Delaware).

**Figure 3:**
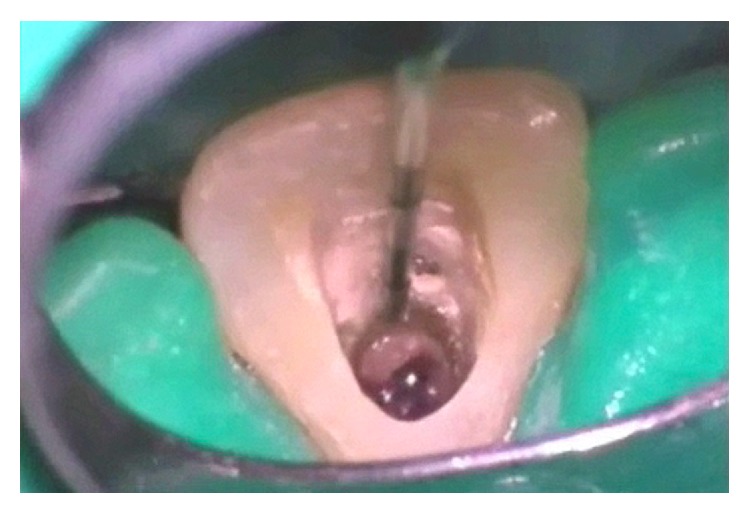
The massive root defect was located 15 mm below the incisal edge.

An access cavity preparation was made on the palatal surface of tooth 21 under magnification with an operative microscope (Opmi Pico, Carl Zeiss Meditec, Oberkochen, Germany). Upon direct examination of the root defect, the diagnosis of buccal root perforation was confirmed (Figure 3). Decision to attempt a nonsurgical root canal treatment and repair of the perforation was made after informing the patient of the different treatment options.

The root canal was shaped manually according to the Schilder technic [17] with copious amounts of 3% sodium hypochlorite solution (Parcan, Septodont, Saint Maur-des-Fossés, France) between each instrument use. Once the cleaning and shaping were completed, the site of the perforation was cleaned with an ultrasonic tip (ET 18D, Actéon, Mérignac, France) under sterile water spray.

After cone fitting, a gutta-percha cone devoid of sealer was seared off apical to the level of the perforation and the gutta-percha was warm molded to the dentinal walls in order to enclose the canal path and prevent inadvertent falling of the cement inside the canal.

**Figure 4:**
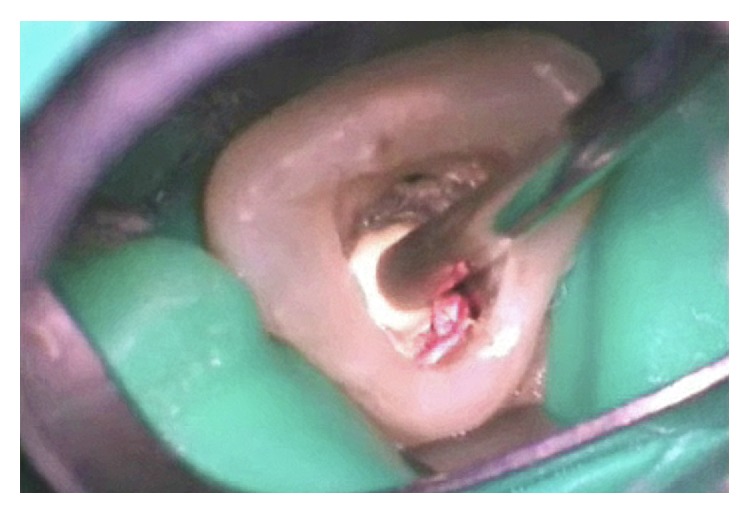
Filling with MTA after enclosing the canal path with gutta-percha.

A mixture of MTA powder (ProRoot MTA, Dentsply, Tulsa, Oklahoma) with sterile water was prepared on a sterile glass slab according to the ratio recommended by the manufacturer (0.33 liquid/powder ratio). A first amount of the mixture was carried with a Messing Gun (Endo Syringe Messing, Produits Dentaires SA, Vevey, Switzerland) inside the root canal and adapted to the defect with a specially designed spatula (W1/2 instrument, West Perforation Repair, Sybron Endo, Orange, California), the excess of moisture was blotted with the blunt tip of a sterile paper point in order to stabilize the cement in situ, and the defect was completely filled in successive increments of material (Figure 4). The tip of a paper point was cut off and moistened with sterile water before being placed in contact with the dampened cement and the access cavity was sealed with a temporary filling (Cavit, 3M ESPE, Saint Louis, MN). The patient was dismissed and another appointment 48 hours later was given.

Tooth isolation and field disinfection were performed as described previously. The temporary dressing was removed as the paper points; the hard setting of the MTA cement was verified with an endodontic probe. After removal of the root canal plug of gutta-percha, the canal was irrigated with 1 mL of a 17% EDTA solution left in place for 1 minute (Largal Ultra, Septodont, Saint Maur-des-Fossés, France) before a final flush with 6 mL of sodium hypochlorite (Parcan, Septodont, Saint Maur-des-Fossés, France) for 5 minutes. After cone fitting, the canal was filled according to the Schilder technic of warm vertical condensation [18] for the down pack using gutta-percha (Autofit, Sybron Endo, Orange, CA) and sealer (Pulp Canal Sealer EWT, Sybron Endo, Orange, CA). The canal was backfilled with injected thermoplasticized gutta-percha (Obtura II, Obtura Spartan, Fenton, MO) below the level of the alveolar crest.

**Figure 5:**
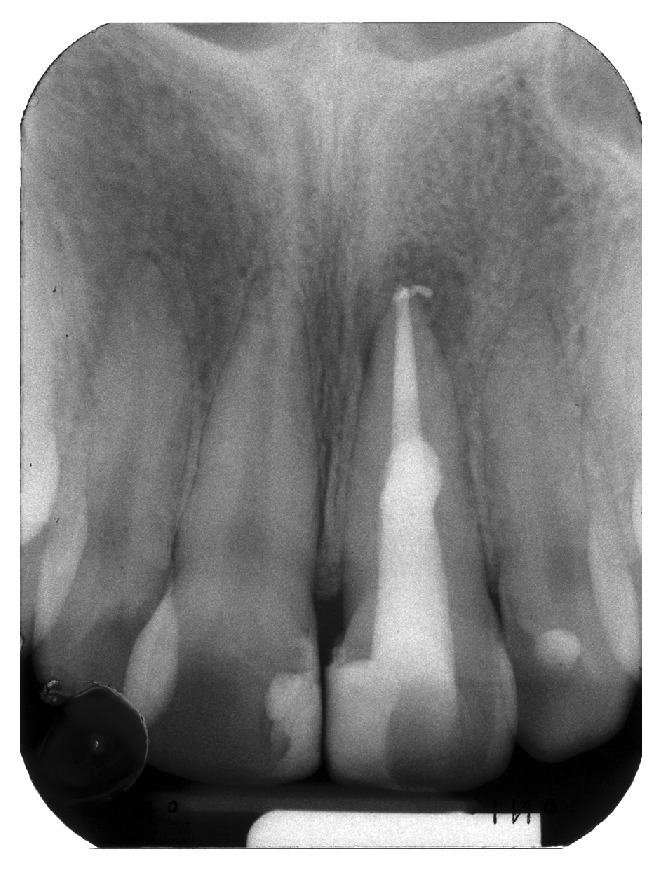
Postoperative radiograph.

The access cavity was filled with a glass ionomer cement (Ketac Fil, 3M ESPE, Saint Louis, MN) covered with a resin composite restoration (EsthetX, Dentsply Caulk, Milford, Delaware) (Figure 5).

**Figure 6:**
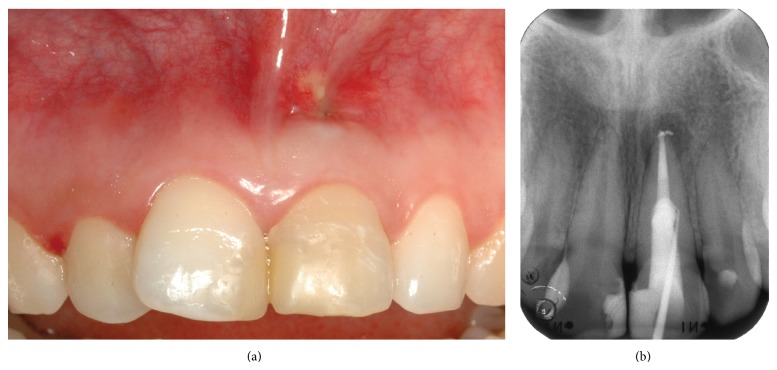
(a) Discoloration of the mucosa at the site of the perforation. (b) Radiograph showing the persistence of the coronal fistula.

At the recall appointment three weeks later, tooth 21 was completely asymptomatic, the fistulous tracts had disappeared, but a scar corresponding to the coronal fistulous tract remained. At review after three months radiographic examination showed that the periapical lesion had reduced in size but a small fenestration of the attached gingiva was present communicating with the perforation site as shown by the exploration with a periodontal probe. Moreover, there was a mucosal discoloration resulting from the MTA placement (Figures 6(a) and 6(b)).

**Figure 7:**
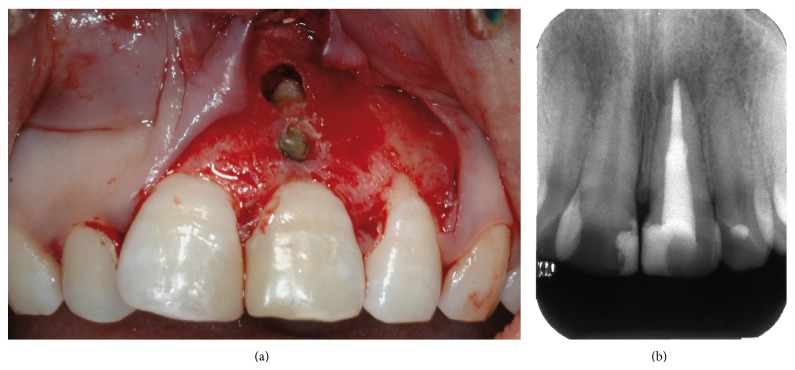
(a) Curettage of the lesions after raising the split thickness flat. (b) Radiograph taken during the surgery.

**Figure 8:**
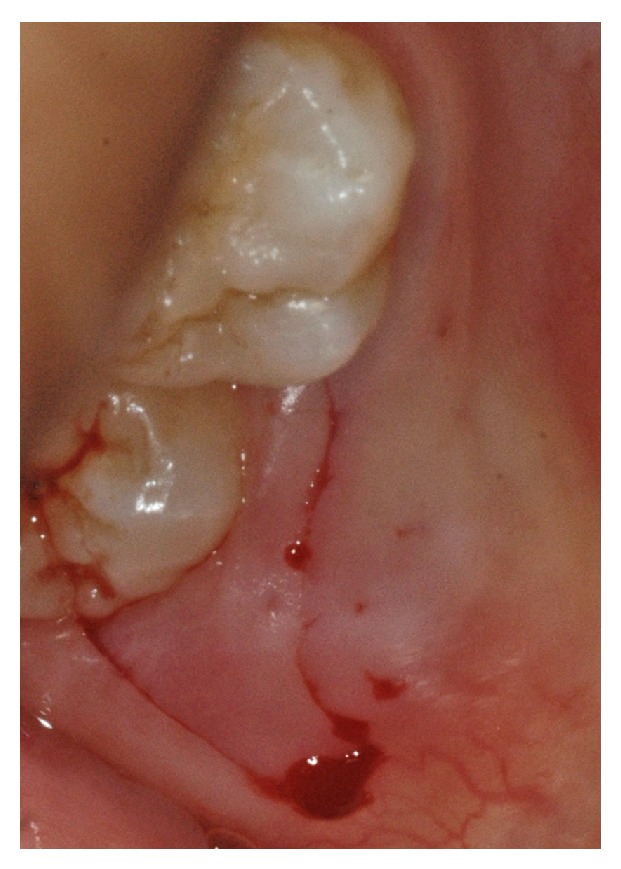
Incision of the tuberosity donor site.

**Figure 9:**
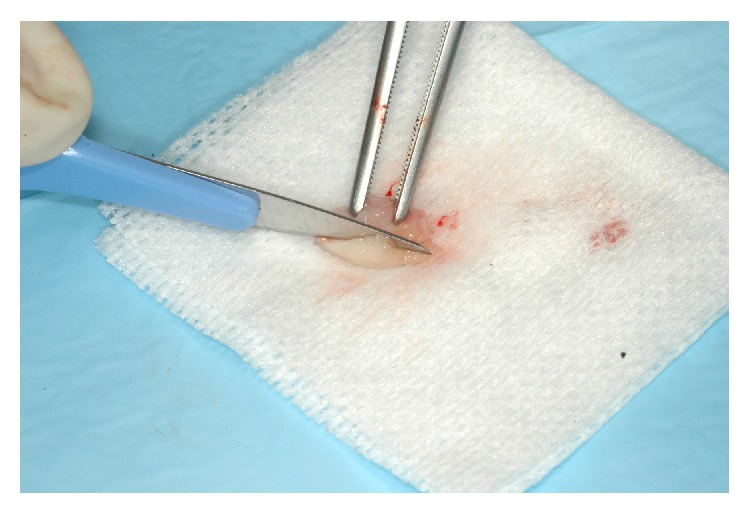
Dissection of the graft to remove epithelium.

**Figure 10:**
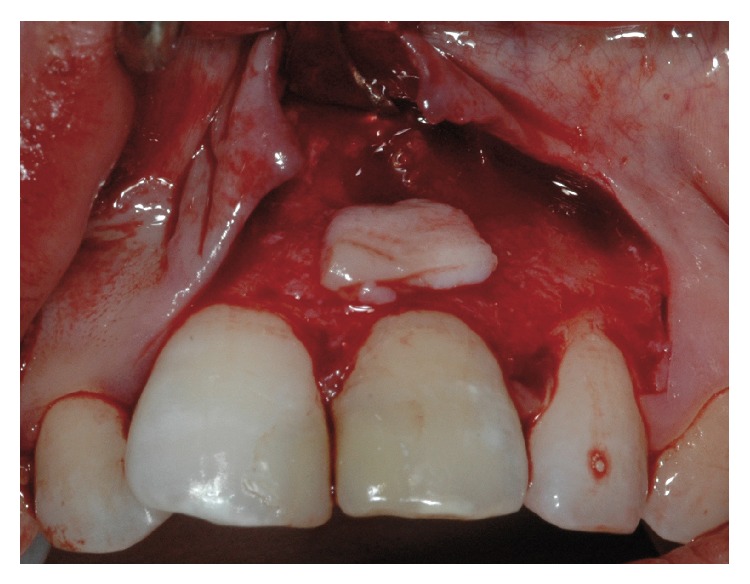
Positioning of the connective graft over the MTA.

The decision was made to perform a surgical correction of the defect and to increase the thickness of the gingiva in order to correct the discoloration. After local anesthesia with 1.6 mL of 4% articaine solution associated with 1/100.000 epinephrine (Septanest, Septodont, Saint Maur-des-Fossés, France), the margin of the fistulous tract was removed by a sharp dissection. A buccal intrasulcular incision, including one tooth mesial and distal of the lesion, was made. A vertical release incision was performed in the distal of tooth 12 extending beyond the mucogingival limit. A split thickness flap, using a 15 blade, was elevated to fully expose the buccal surface of the root. The fibrous, adhering tissues over the root were carefully removed. After raising the flap, it was possible to confirm that the periapical lesion did not communicate with the fenestration even if it had also perforated the facial cortical plate and that a small ridge of crestal bone remained coronally to the perforation. An apical curettage of the periapical lesion was performed in order to remove any extruding sealer from the previous root canal filling and to promote healing on both inflammatory sites. Neither apicoectomy nor retrofill was done since nonsurgical root canal treatment following root repair led to the disappearance of the most apical fistulous tract (Figures 7(a) and 7(b)). The slight overfill of material exceeding the root perforation defect was removed with a fine grit diamond bur under saline solution irrigation. A connective tissue graft was harvested from the right maxillary tuberosity using a 12 blade (Figure 8). The wound closure was made with 4/0 vicryl sutures (Ethicon, Auneau, France). The graft was split and the epithelium was removed (Figure 9). The connective tissue graft was positioned to cover the entire defect and was sutured to the pedicle flap (Figure 10). The flap was repositioned and sutured with interrupted 5/0 vicryl sutures (Ethicon, Auneau, France). A postoperative nonsteroidal anti-inflammatory drug prescription was given to the patient with instructions to take 200 mg of ibuprofen every 6 hours for four days, to perform antiseptic mouth rinses three times per day for one week and to avoid brushing of the upper front teeth for the next five days. The sutures were removed the seventh day after surgery.

**Figure 11:**
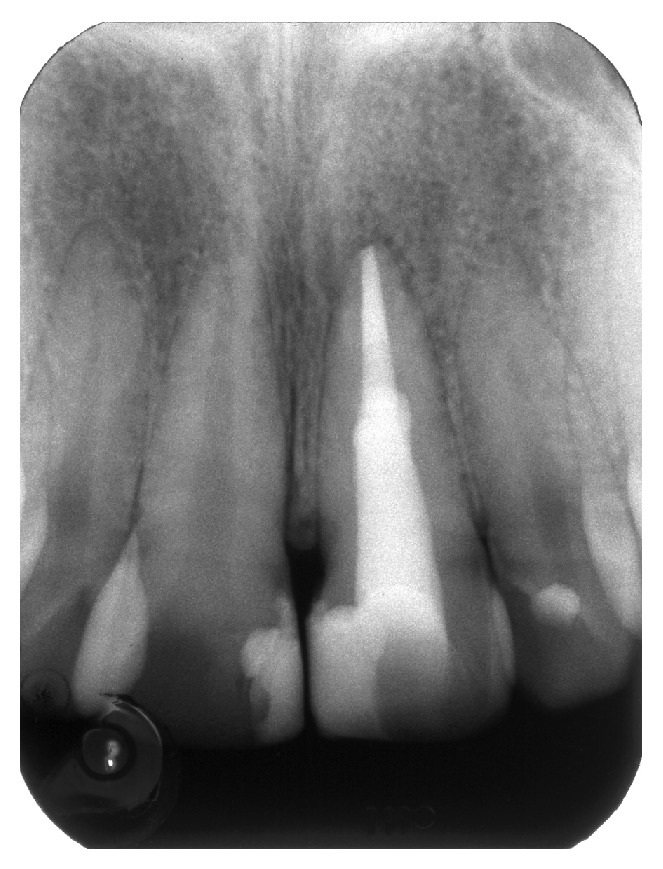
Radiograph at 3 months after surgery.

**Figure 12:**
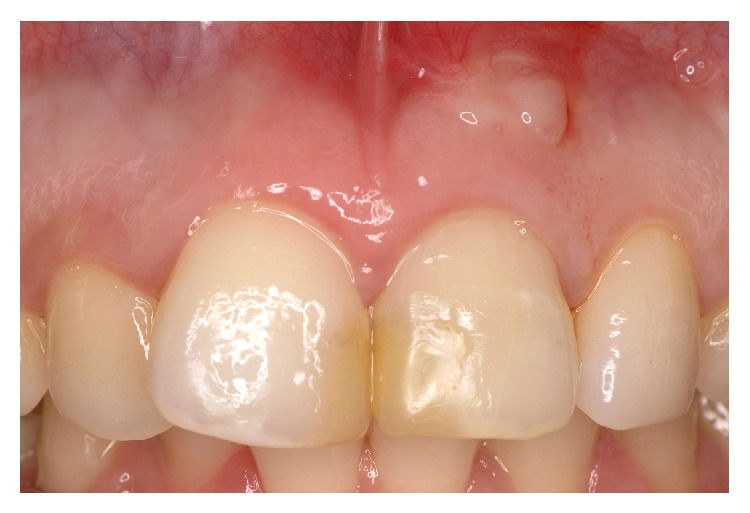
Scar tissue formation at the site of the connective graft.

The six-month follow-up of the case did not reveal any recurrence of mucosal fenestration and gingival healing appeared complete. Probing depths on labial surface were within normal limit. The periapical lesion was in the course of healing (Figure 11). At this stage the patient complained of a slight tooth discoloration of tooth 21 and of a visible scar on the graft site (Figure 12). For a better esthetic result, it was decided that a tooth whitening procedure would be performed as well as a peeling of the graft site.

**Figure 13:**
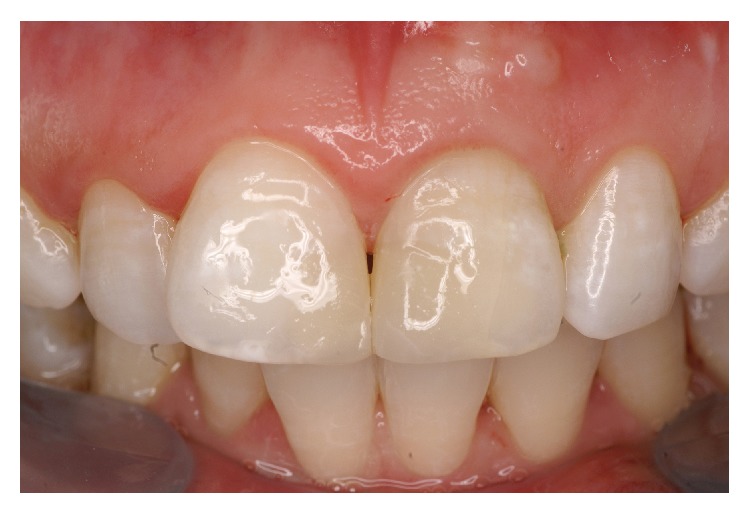
Aesthetic result achieved after peeling, teeth whitening, and composite refection.

A partial-thickness incision was performed under local anesthesia, using a 15 blade, to remove the prominent part of the graft. As in an external bevel gingivectomy, no suturing or dressing was used leaving a connective tissue exposure, and hemostasis was achieved by means of compression with a piece of sterile moist gauze. That patient was asked to use antiseptic mouthwash twice a day for 7 days (0.12% chlorhexidine). After secondary intention healing of the gingival wound, an intracoronal tooth whitening procedure was first performed on tooth 21 in two separate appointments according to the standard walking bleach technic [19], before vital bleaching of both arches. Total amount of time for both procedures was two weeks and the improvement in tooth whitening was noticeable using the Vita Classical shade guide (from Vita shade A3.5 to A2). The access cavity of tooth 21 was filled three weeks after completion of the bleaching procedure in order to let the dentinal substrate recover its oxygen-free condition before definitive restoration [20]. At this stage all-composite material was removed on both teeth 11 and 21 and replaced with new composite build-ups (CeramX Duo, Dentsply DeTrey GmbH, Konstanz, Germany) according to a simplified stratification protocol [21] (Figure 13).

**Figure 14:**
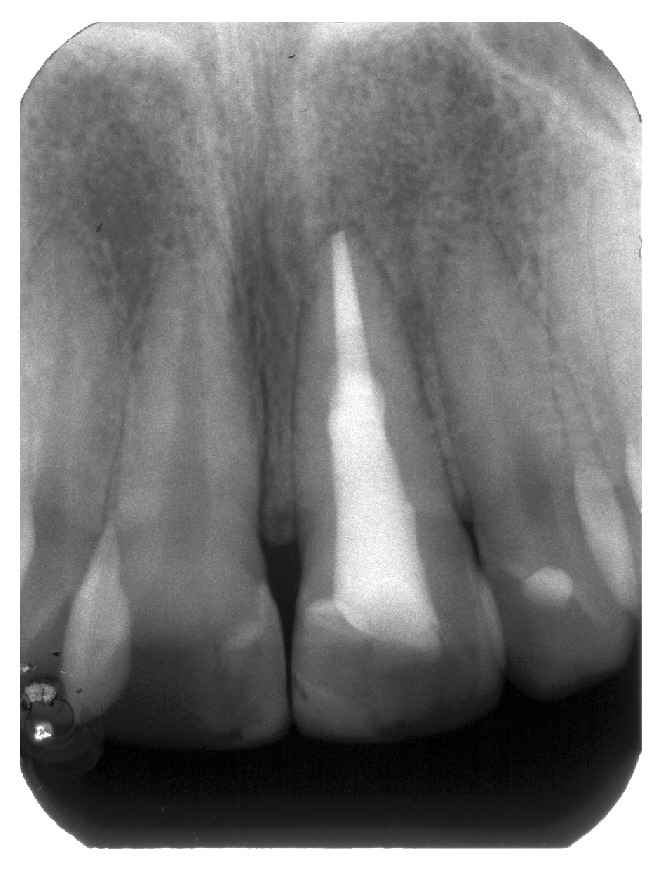
Radiograph at 1 year.

At the one-year follow-up, the gingiva maintained its healthy state. The periapical lesion was still healing (Figure 14).

**Figure 15:**
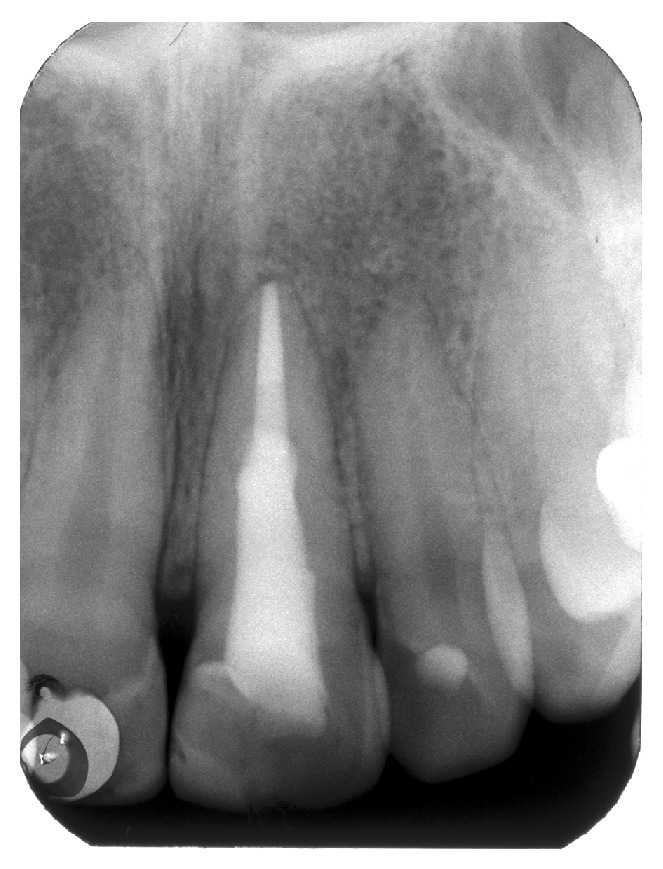
Radiograph at 2 years.

**Figure 16:**
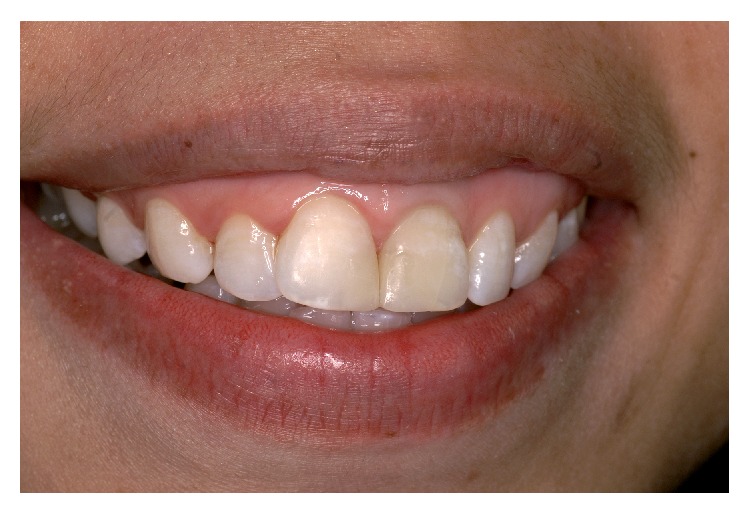
Stability of the result at 2 years.

At the two-year follow-up, the periapical lesion had completely healed and was asymptomatic (Figure 15); the patient remained satisfied with its appearance (Figure 16).

## 3. Discussion

The case presented in this paper was a severely compromised central maxillary incisor. The site of the perforation was several millimeters beyond the crest; the wide area of the communication with the external surface of the root and thinning of the root canal wall made the prognosis poor. Treatment options were discussed with the patient, including immediate extraction and subsequent restoration with either a three-unit partial fixed prosthesis or a single-tooth implant. Even if an analytic framework could theoretically help in choosing the option the most likely to succeed, clinical decision can only be made at the patient level taking into account individual perception of success and financial considerations [22, 23]. Considering the absence of periodontal pockets, the prognosis, and the cost-effectiveness of each procedure, it was decided to try to retain the tooth and to attempt a nonsurgical root canal treatment with reparation of the perforation with a calcium silicate cement.

The extensive mucosal and osseous damage the patient came in with incited us to first repair the root defect instead of placing an interappointment calcium hydroxide dressing to obtain the fistula's closure. Our aim in this procedure was to prevent further periodontal destruction, especially in a coronal direction to avoid downgrowth of gingival epithelium to the level of the perforation site. Furthermore, cases of gingival necrosis have been reported with calcium hydroxide extrusion into surrounding tissues [24, 25].

Bargholz [26] described the use of a bioresorbable barrier to form an internal matrix against which the sealing material could be condensed. Because of the infectious condition of the perforation site and its communication with the oral cavity, we did not use such a barrier. The size of the root perforation, its unfavorable configuration, and the absence of natural external matrix due to the fenestration of mucosa and bone were contributing factors that explained the difficulty of confining repair material inside the root.

Due to the localization of the osseous defect below the level of the mucogingival junction and the thinness of the free gingiva, the healing process resulted in a mucosal fenestration and discoloration of this soft tissue area. Discoloration caused by MTA placement has been previously reported in literature and was related to iron oxide [27, 28]. White MTA was used in this case instead of grey MTA in order to prevent the problem. But in spite of this, setting of the cement in the contact with blood caused the coloration of the material to turn grey. Even if contamination of the cement mix with moisture or blood does not seem to affect the final properties of this particular material, we could not exclude that the grey discoloration originated from the degradation of blood pigments. The patient had a high smile line and consequently the localisation of the coloration near the mucogingival junction was visible when smiling.

The prognosis of perforations, which do communicate with the oral cavity, is considered to be questionable [29]. Guided tissue regeneration has been attempted to address fenestration or dehiscence by serving as a barrier for apical migration of the gingival epithelium [29], but the technic is both expensive and operator dependant. In this case, the patient had a healthy periodontium without periodontal pockets. Furthermore, the osseous defect associated with the perforation does not communicate with the periapical lesion. Tobon-Arroyave used a periosteal graft to close a chronic sinus tract associated with a periapical and marginal lesion [30]. This technic would have probably ensured the closure of the fistula, but the discoloration might have remained after healing because of the absence of thickening of the soft tissue. Russo used a free gingival graft to cover an amalgam tattoo [31]. This technic can also be used in the treatment of mucogingival fenestration [32]. For better esthetic results and to ensure a complete closure of the mucosal fenestration, we chose to correct the defect with a connective tissue graft. This technic has been previously described to cover a resin ionomer cement in the treatment of invasive cervical root resorption [33] or root perforation [34]. Harris described a case of plastic surgery with connective tissue graft to replace missing periodontal structures after repair of a coronal root perforation with silver amalgam [35]. The choice of the tuberosity as the donor site was made because of the thickness of the tissue and the low postoperative morbidity. This harvesting technic is frequently used in mucogingival surgery and provides good results for root coverage [36]. In case of mucosal fenestration, the difference of colour of the donor site and the recipient site has been previously described with palatal subepithelial connective tissue graft [37].
